# Lung function and frailty: Dose–response relationship, mediation effects, and prediction model

**DOI:** 10.1097/MD.0000000000044529

**Published:** 2025-09-12

**Authors:** Sirui Zhou, Ping Wang, Weijian Zhu, Yulan Zeng

**Affiliations:** a Department of Respiration, Liyuan Hospital, Tongji Medical College, Huazhong University of Science and Technology, Wuhan, Hubei Province, China; b Department of Respiratory and Critical Care Medicine, Chest Hospital of Tianjin University, Tianjin, China; c Department of Orthopaedics, Liyuan Hospital, Tongji Medical College, Huazhong University of Science and Technology, Wuhan, Hubei Province, China.

**Keywords:** cognition, frailty, logistic regression, peak expiratory flow, predictive model

## Abstract

Lung function, cognitive ability, and lifestyle are key factors affecting the health of older adults, especially in predicting frailty. However, the relationship between lung function and frailty is unclear, particularly in Chinese middle-aged and elderly populations, and clinical frailty assessment methods lack standardization. This study aimed to develop a frailty risk prediction model by analyzing lung function, cognitive ability, and lifestyle factors, providing a basis for early frailty screening and intervention. Data from the baseline wave (2011–2012) of the China Health and Retirement Longitudinal Study were used, including 6065 individuals aged ≥ 45 years. Peak expiratory flow (PEF) was measured using standardized procedures. The percentage of predicted PEF (PEFpred%) was calculated as (measured PEF/predicted PEF) × 100%, where predicted values were estimated based on age-, sex-, and height-adjusted reference equations derived from a Chinese population. Frailty was assessed through a questionnaire, and relevant covariates were recorded. Multilevel logistic regression analyzed the relationship between PEFpred% and frailty, with Bootstrap methods evaluating the mediating role of cognitive function. Least absolute shrinkage and selection operator regression and cross-validation were used to identify key predictors for the frailty risk model, evaluated by receiver operating characteristic and calibration curves. The mean age of the participants was 58.3 years, with 51% female. Twenty-three point five percent had a PEFpred% <60%. Higher PEFpred% was associated with lower frailty risk. Dose–response analysis showed a nonlinear relationship, with significant risk increases when PEFpred% was < 80.03%. Cognitive function partially mediated the relationship, explaining 20.11% of the effect. The model based on PEFpred%, cognitive function, and other variables showed good performance: area under the curve was 0.796 in the training set and 0.775 in the validation set. PEFpred% is a useful predictor of frailty risk in Chinese middle-aged and elderly individuals, with cognitive function playing a key mediating role. The frailty risk prediction model demonstrates good performance and warrants further clinical validation.

## 1. Introduction

As the global aging process accelerates, China now has the largest elderly population in the world. Data from the National Bureau of Statistics of China indicates that, by the end of 2023, there were 297 million individuals aged 60 and older, making up 21.1% of the total population.^[1]^ This demographic shift poses significant challenges for the healthcare system.^[2]^ Frailty, a term used in a biological context, refers to a state of increased vulnerability characterized by declines in strength, endurance, and physiological function.^[3,4]^ As older adults age, the likelihood of frailty increases due to declines in physical, mental, and cognitive functioning, along with a heightened risk of chronic diseases.^[5,6]^ Furthermore, frailty is significantly associated with adverse health outcomes, such as falls, fractures, and disabilities, which can adversely affect individuals, families, and society as a whole.^[7]^ Currently, the dominant methods for assessing frailty are the frailty phenotype and the frailty index.^[8]^ The fried frailty phenotype assessment identifies frailty based on 5 criteria: unintentional weight loss, self-reported fatigue, lethargy, slow walking speed, and low physical activity. Individuals are categorized as frail if they meet 3 or more of these criteria.^[9]^ Additionally, pre-frailty is recognized as a transitional phase between robust health and clinical frailty, indicating that these individuals are at risk of transitioning to frailty. Internationally, the prevalence of frailty among the elderly ranges from approximately 4% to 10%, with reported rates of 9.4% in Australia, 8.1% in the United Kingdom, 7.4% in Canada, 7.0% in France, and 6.5% in Italy.^[10]^ In contrast, a previous meta-analysis conducted in a Chinese community indicated that the prevalence of frailty and pre-frailty among the elderly was 10% and 43%, respectively. Key independent risk factors for frailty in this population included advanced age, female gender, and the presence of 3 or more chronic diseases.^[7]^

Pulmonary function testing provides an objective and repeatable method for assessing airway airflow limitation. Patients with chronic lung conditions, like chronic obstructive pulmonary disease, asthma, and lung cancer, are often evaluated through pulmonary function tests to gauge respiratory function and the effectiveness of treatments. In the general population, respiratory function may also serve as a significant predictor of future health outcomes.^[11]^ Previous studies have indicated that impaired respiratory function is linked to an increased risk of cardiovascular disease, dementia, disability, and mortality in older adults, suggesting that it may serve as a marker of frailty and its consequences.^[12–14]^ While spirometry is the gold standard for measuring lung function, its use is often impractical in austere environments due to the high demands on equipment and personnel.^[15]^ In contrast, peak expiratory flow (PEF), a core parameter of spirometry, measures the highest flow rate during forceful expiration and is typically assessed using a peak flow meter.^[16]^ Although PEF is slightly less accurate than spirometry, it is easier to perform, cost-effective, and can be widely utilized in primary care settings.^[17]^ In this context, our study aims to enhance the understanding of the relationship between lung function and frailty. Early detection of pre-frail individuals, coupled with appropriate interventions, can help mitigate the burden on healthcare organizations and society by potentially reversing the pre-frail state back to health.^[18]^

## 2. Methods

### 2.1. Data sources

The China Health and Retirement Longitudinal Study (CHARLS) database comprises a randomized sample of individuals aged 45 and older, drawn from 450 villages across 150 counties and districts in 28 provinces in China. The study aims to assess associations between health and socio-economic factors.^[19]^ CHARLS received ethical approval from the Ethical Review Board of Peking University (IRB00001052–11015), and all participants gave their written informed consent. This study utilizes de-identified CHARLS data. As the analysis involved secondary processing of preapproved anonymized datasets, no additional ethics review was required.

**Figure 1.**
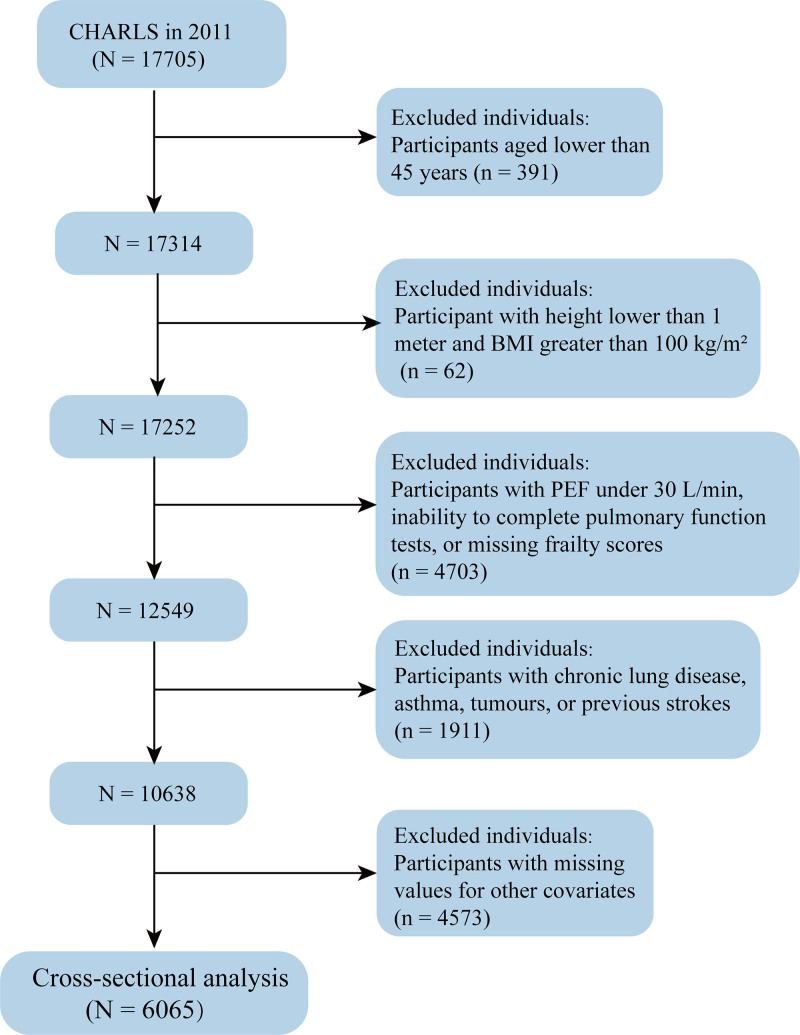
Flowchart of participant selection for this study.

This study utilized baseline data from the CHARLS database collected between May 2011 and March 2012, including information obtained through questionnaires and blood tests. Individuals aged 45 years and older at baseline were eligible for inclusion. To ensure data quality and reduce potential confounding, participants were excluded based on the following criteria: height ≤ 1 m or body mass index (BMI) ≥ 100 kg/m², as such implausible anthropometric values may reflect measurement error or data entry inaccuracies; PEF < 30 L/min or inability to complete pulmonary function testing, indicating unreliable or nonstandard measurements; a history of lung disease or asthma, which independently affect both PEF and frailty; a diagnosis of cancer, due to its potential influence on frailty through systemic burden or treatment-related decline; a history of stroke, given its impact on both cognitive and physical function; and missing data on essential covariates, which would compromise the validity of statistical analysis. Details on the proportion of missing data for each covariate are provided in Table S1, Supplemental Digital Content, https://links.lww.com/MD/P953. The participant selection process is illustrated in Figure 1.

### 2.2. Data collection and design

#### 2.2.1. Peak expiratory flow rate

The measurement of PEF followed a standardized procedure: the subject stood upright, inhaled deeply until the lungs were fully inflated, held their breath, then placed the mouthpiece completely in their mouth and exhaled as forcefully and quickly as possible. To ensure data accuracy, the highest PEF value obtained from 3 measurements was recorded as the final result. Predicted PEF values for individuals of different genders were calculated using the Zhong Nanshan equation for Chinese adults^[20]^:


$$\begin{array}{lll} MalePEF(L/min): & 75.6+20.4*age-0.41*{age}^{2} & +0.002*{age}^{3}+1.19*height. \end{array}$$

$$\begin{array}{lll} FemalePEF(L/min): & 282.0+1.79*age-0.046*{age}^{2} & +0.68*height. \end{array}$$

Since PEF values are influenced by gender, age, and height, the ratio of the actual PEF value to the predicted value, expressed as PEFpred%, is typically used as a standardized criterion for result interpretation.^[16]^ A PEFpred% of ≥ 80% indicates no expiratory airflow limitation, a PEFpred% between 60% and 79% suggests mild limitation of expiratory function, and a PEFpred% of < 60% indicates severe restriction of expiratory function.^[16]^ This grading criterion was applied in the present study, categorizing PEFpred% into 3 groups (Q1, Q2, and Q3) from low to high for analysis.

#### 2.2.2. Frailty

Adaptation of frailty assessment criteria from published literature in conjunction with the CHARLS database^[21]^:

1. Weakness was deemed present if the participant reported difficulty lifting or carrying heavy objects weighing more than 5 kg.
2. Slowness was determined if the participant indicated difficulty walking 100 m or climbing multiple flights of stairs without taking breaks.
3. Emotional or physical exhaustion was identified if the participant selected either “most or all of the time” or “occasionally to moderately all of the time” for the relevant item on the Chinese version of the Centre for Epidemiological Studies Depression Scale questionnaire.
4. Participants were classified as having low physical activity if they reported not engaging in any physical activity during the usual week or if they walked for less than 10 minutes at a time.
5. Weight loss was considered present if the participant had inadvertently lost 5 kg or more within the last year or had a current BMI of ≤ 18.5 kg/m².

Scores for the above criteria were calculated such that an individual score of 0 indicated no frailty, a score of 1 to 2 categorized the individual as pre-frail, and a score of 3 or higher classified them as frail.

#### 2.2.3. Covariate variables

In this study, we analyzed a range of covariates that may influence lung function and frailty status, including demographic information, lifestyle habits, medical history, physical examination results, and questionnaire responses.

Demographic information: this included age, gender, marital status, place of residence, education level, height, weight, and BMI. Gender was categorized as “male” and “female.” Marital status was classified as “married and cohabiting” or “single,” with the latter encompassing those who are separated, divorced, widowed, or never married. Residence was categorized as “rural” or “urban.” Educational attainment was divided into 4 categories: “illiterate” (no education), “primary education” (kindergarten and primary), “secondary education” (lower and upper secondary), and “higher education” (university and above). Lifestyle habits: smoking and alcohol consumption were categorized based on current or past habits, while sleep duration was recorded as the average daily sleep time reported in the questionnaire. Medical history: This included conditions such as hypertension, diabetes, heart disease, mental illness, gastric disease, liver disease, kidney disease, and arthritis. Hypertension was defined as either a prior diagnosis or a current systolic blood pressure ≥ 140 mm Hg and/or diastolic blood pressure ≥ 90 mm Hg. Diabetes was defined as a previous diagnosis or random blood glucose ≥ 11.1 mmol/L or HbA1c ≥ 6.5%. Physical examination indices: we measured total cholesterol, triglycerides, low-density lipoprotein, high-density lipoprotein, HbA1c, C-reactive protein, and the triglyceride glucose index (TyG) along with the triglyceride-glucose body mass index (TyG-BMI). TyG and TyG-BMI serve as indices for assessing insulin resistance, with their calculation methods detailed in previous studies.^[22]^

In addition, cognitive function scores derived from the questionnaire were included in the analysis of covariates. The cognitive functioning assessment encompassed 4 major domains: everyday memory, vocabulary memory, arithmetic skills, and graph drawing, comprising a total of 21 specific tests.^[23]^ The everyday memory assessment focused on the individual’s recognition of the current time and season. In the vocabulary memory test, the assessor read out 10 words randomly, and participants were required to recall as many words as possible; a higher number of recalled words indicated a better score. The arithmetic test involved performing 5 consecutive subtractions of 7 from 100 to evaluate calculation skills. The graphing component required participants to accurately copy a provided graph, with 1 point awarded for correct completion. Each test employed a binary scoring system: a correct response received 1 point, while an incorrect response received 0 points, with higher scores reflecting greater cognitive ability.^[24]^

### 2.3. Data analysis

In this study, we applied one-way analysis of variance for continuous variables that satisfied normal distribution and the Kruskal–Wallis rank sum test for those that were not normally distributed. Categorical variables were analyzed using the χ² test to ensure consistency and comparability of baseline data distribution. Descriptive statistics for continuous variables were expressed as mean ± standard deviation (mean ± SD), while categorical variables were presented as counts and corresponding percentages.

To explore the relationship between PEFpred% and frailty scores, we constructed a 3-level progressive logistic model. Model 1 was the crude model, unadjusted for any variables. Model 2 was adjusted for age, gender, BMI, marital status, education, place of residence, sleep duration, smoking, and alcohol consumption, representing key demographic and lifestyle factors. Model 3 was the fully adjusted model, incorporating all covariates included in the study to control for confounding effects. We then utilized 4 restricted cubic splines based on Model 3 to analyze the dose–response relationship between PEFpred% and frailty.

Additionally, we introduced cognitive function as a mediating variable and conducted 1000 resampling trials using the nonparametric Bootstrap method.^[25]^ This approach aimed to accurately measure the indirect effect of PEFpred% on frailty status through cognitive function, as well as the direct effect after excluding the mediating influence, thereby assessing the relative weights of the overall, indirect, and direct effects.^[26]^

Finally, we illustrated the relationships among PEFpred%, cognitive function, and frailty using column-line plots. We employed the least absolute shrinkage and selection operator regression analyses to construct a predictive model for frailty risk. The sample was randomly divided into training and validation groups, with analyses conducted on the training set and significant predictors identified through 10-fold cross-validation. These predictors were included in multifactor logistic regression analyses, with models constructed based on a *P*-value threshold of <.05. We validated the model using both training and validation groups, assessing predictive ability through the area under the curve (AUC) of the receiver operating characteristic (ROC) analysis. Calibration curves were used to analyze the consistency between predicted probabilities and actual outcomes.

All data analyses and visualizations were performed using RStudio software (version 4.3.3, Posit Software, Boston), utilizing packages such as “tableone,” “stats,” “rcs,” “dplyr,” “mediation,” “glmnet,” “rms,” and “pROC.” Statistical significance was determined at *P* < .05.

## 3. Results

### 3.1. Baseline population data

The baseline data for the 6065 subjects included in this study are summarized in Table 1. When categorized by frailty status (none, pre-frailty, and frailty), the population had a mean age of 58.3 ± 8.7 years, a BMI of 23.8 ± 3.9 kg/m², a sleep duration of 6.4 ± 1.8 hours, a cognitive function score of 12.0 ± 3.5 points, a glycated hemoglobin level of 5.3 ± 0.8%, and a PEFpred% of 80.5 ± 26.7%.

**Table 1:** Characteristics of the participants at baseline (N = 6065).

	Level	Overall	None	Per-frailty	Frailty	*P*
n		6065	2742	3054	269	
Age (mean [SD])		58.3 (8.7)	57.3 (8.4)	58.7 (8.8)	63.1 (9.7)	<.001
Gender (%)	Female	3092(51.0)	1265 (46.1)	1656 (54.2)	171 (63.6)	<.001
	Male	2973(49.0)	1477 (53.9)	1398 (45.8)	98 (36.4)	
Marital status (%)	Single	613 (10.1)	218 (8.0)	351 (11.5)	44 (16.4)	<.001
	Married	5452 (89.9)	2524 (92.0)	2703 (88.5)	225 (83.6)	
Education (%)	Illiterate	1324 (21.8)	492 (17.9)	734 (24.0)	98 (36.4)	<.001
	Primary	2573 (42.4)	1096 (40.0)	1359 (44.5)	118 (43.9)	
	Middle school	2064 (34.0)	1085 (39.6)	927 (30.4)	52 (19.3)	
	Tertiary	103 (1.7)	69 (2.5)	33 (1.1)	1 (0.4)	
BMI (mean [SD])		23.8 (3.9)	24.2 (3.7)	23.5 (4.0)	23.0 (4.6)	<.001
Residence (%)	Rural	2280 (37.6)	1112 (40.6)	1087 (35.6)	81 (30.1)	<.001
	Urban	3785 (62.4)	1630 (59.4)	1967 (64.4)	188 (69.9)	
Drinking (%)	No	3466 (57.1)	1486 (54.2)	1804 (59.1)	176 (65.4)	<.001
	Yes	2599 (42.9)	1256 (45.8)	1250 (40.9)	93 (34.6)	
Smoking (%)	No	3627 (59.8)	1577 (57.5)	1878 (61.5)	172 (63.9)	.003
	Yes	2438 (40.2)	1165 (42.5)	1176 (38.5)	97 (36.1)	
Hypertension (%)	No	3200 (52.8)	1448 (52.8)	1636 (53.6)	116 (43.1)	.004
	Yes	2865 (47.2)	1294 (47.2)	1418 (46.4)	153 (56.9)	
Diabetes (%)	No	5175 (85.3)	2363 (86.2)	2606 (85.3)	206 (76.6)	<.001
	Yes	890 (14.7)	379 (13.8)	448 (14.7)	63 (23.4)	
Cardiovascular disease (%)	No	5396 (89.0)	2507 (91.4)	2683 (87.9)	206 (76.6)	<.001
	Yes	669 (11.0)	235 (8.6)	371 (12.1)	63 (23.4)	
Psycho (%)	No	6006 (99.0)	2728 (99.5)	3012 (98.6)	266 (98.9)	.004
	Yes	59 (1.0)	14 (0.5)	42 (1.4)	3 (1.1)	
Arthritis (%)	No	4096 (67.5)	2037 (74.3)	1940 (63.5)	119 (44.2)	<.001
	Yes	1969 (32.5)	705 (25.7)	1114 (36.5)	150 (55.8)	
Stomach disorders (%)	No	4761 (78.5)	2263 (82.5)	2308 (75.6)	190 (70.6)	<.001
	Yes	1304 (21.5)	479 (17.5)	746 (24.4)	79 (29.4)	
Liver disease (%)	No	5884 (97.0)	2679 (97.7)	2950 (96.6)	255 (94.8)	.004
	Yes	181 (3.0)	63 (2.3)	104 (3.4)	14 (5.2)	
Chronic kidney disease (%)	No	5744 (94.7)	2630 (95.9)	2866 (93.8)	248 (92.2)	<.001
	Yes	321 (5.3)	112 (4.1)	188 (6.2)	21 (7.8)	
Cognition (mean [SD])		12.0 (3.5)	12.7 (3.3)	11.5 (3.4)	9.8 (3.6)	<.001
Sleep time (mean [SD])		6.4 (1.8)	6.6 (1.7)	6.4 (1.9)	5.6 (2.3)	<.001
TC (mean [SD])		193.4 (37.6)	193.3 (37.7)	193.8 (37.4)	191.0 (38.8)	.5
TG (mean [SD])		133.0 (97.5)	133.7 (99.9)	132.7 (97.2)	130.0 (72.3)	.812
HDL (mean [SD])		50.7 (14.9)	50.3 (14.6)	51.2 (15.2)	49.6 (15.0)	.03
LDL (mean [SD])		116.8 (34.6)	117.1 (35.2)	116.6 (34.0)	115.7 (34.9)	.755
HbA1C (mean [SD])		5.3 (0.8)	5.2 (0.8)	5.3 (0.8)	5.4 (1.1)	<.001
CRP (mean [SD])		2.5 (7.4)	2.5 (7.8)	2.5 (7.2)	2.6 (5.1)	.981
TyG (mean [SD])		8.7 (0.7)	8.7 (0.7)	8.7 (0.7)	8.7 (0.6)	.35
TyG_BMI (mean [SD])		207.5 (41.6)	211.0 (40.0)	204.8 (42.1)	202.0 (48.0)	<.001
PEFpred% (group)	Q1	1423(23.5)	567(20.7)	763 (25.0)	93 (34.6)	<.001
	Q2	1561(25.7)	659 (24.0)	829 (27.1)	73 (27.1)	
	Q3	3081(50.8)	1516(55.3)	1462(47.9)	103 (38.3)	
PEFpred% (mean [SD])		80.5 (26.7)	83.6 (26.7)	78.5 (26.4)	71.9 (25.8)	<.001

BMI = body mass index, CRP = C-reactive protein, HDL = high-density lipoprotein, LDL = low-density lipoprotein, PEF = peak expiratory flow, TC = total cholesterol, TG = triglycerides, TyG = triglyceride glucose index.

The analysis indicated that older individuals, females, who had a lower BMI, were married, possessed a lower level of education, resided in rural areas, experienced shorter sleep duration, and exhibited poorer cognitive function, were more likely to be frail. These findings are consistent with previous research in the field.

### 3.2. Dose–response relationship between PEFpred% and frailty

In Table 2, we observe a significant decreasing trend in the odds ratio (OR) for frailty status among middle-aged and elderly individuals as the PEFpred% increases. This trend is evident across all models analyzed, including the basic model (Model 1) and the fully adjusted model (Model 3). Notably, the Q3 group exhibited lower ORs for frailty status compared to the Q1 group in all models, with *P*-values consistently below .05. Specifically, in Models 1 and 2, *P*-values were <.001, indicating a strong negative correlation between higher levels of PEFpred% and a reduced risk of frailty. This reinforces the conclusion that improved pulmonary function is associated with better frailty outcomes in this population.

**Table 2:** Correlation between PEFpred% and frailty risk in CHARLS.

	Model 1		Model 2		Model 3	
	OR (95% CI)	*P*	OR (95% CI)	*P*	OR (95% CI)	*P*
Q1	Ref		Ref		Ref	
Q2	0.92 (0.87–0.98)	.01	0.97 (0.91–1.03)	.26	0.99 (0.93–1.05)	.78
Q3	0.82 (0.78–0.87)	<.001	0.87 (0.83–0.92)	<.001	0.93 (0.88–0.98)	.005
*P* for trend	<.001		<.001		.002	

Model 1 was the crude model, unadjusted for any variables. Model 2 was adjusted for age, gender, BMI, marital status, education, place of residence, sleep duration, smoking, and alcohol consumption. Model 3 was the fully adjusted model, incorporating all covariates in the study to control for confounding effects.

CHARLS = China Health and Retirement Longitudinal Study, CI = confidence interval, OR = odds ratio.

**Figure 2.**
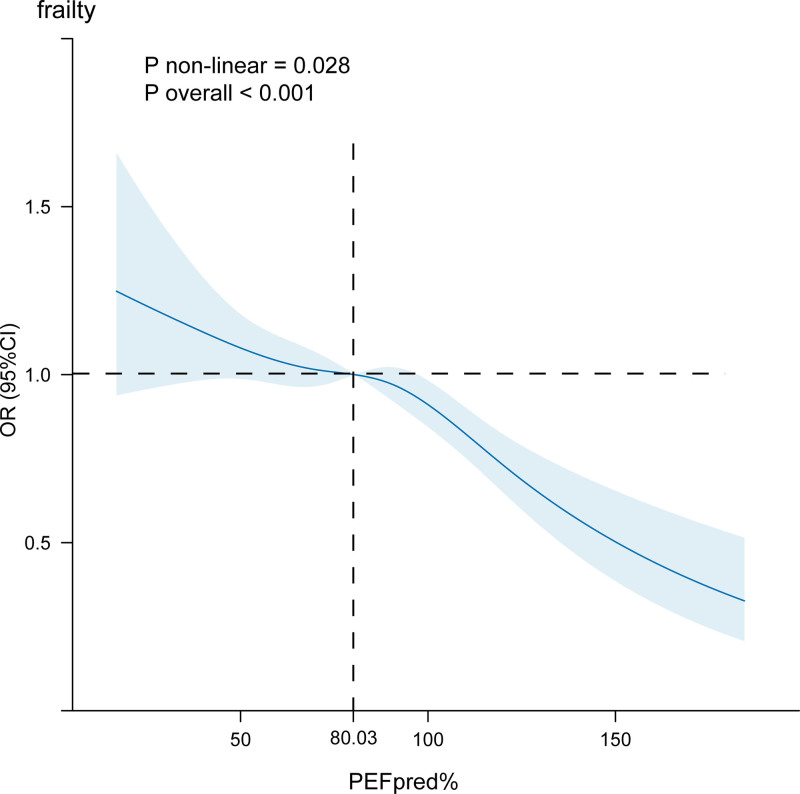
Restricted cubic spline plot of the correlation between PEFpred% and frailty risk. This plot was constructed based on Model 3. The model adjusted for sex, age, marital status, place of residence, education, height, weight, BMI, smoking, alcohol consumption, sleep duration, hypertension, diabetes, heart disease, psychiatric disorders, gastric disorders, liver disorders, renal disorders, arthritis, cholesterol, triglycerides, LDL, HDL, glycated hemoglobin, C-reactive protein, triglyceride glucose index, and triglyceride-glucose body mass index. BMI = body mass index, HDL = high-density lipoprotein, LDL = low-density lipoprotein.

Figure 2 illustrates the dose–response relationship between PEFpred% and frailty risk. The restricted cubic spline plot indicates a nonlinear negative correlation between PEFpred% and the likelihood of frailty, with a *P*-value for non-linearity of .028. Specifically, when PEFpred% falls below 80.03%, there is a marked increase in the risk of frailty among middle-aged and elderly individuals. This finding highlights the critical threshold at which pulmonary function may significantly impact frailty risk in this demographic.

### 3.3. Subgroup analysis

**Figure 3.**
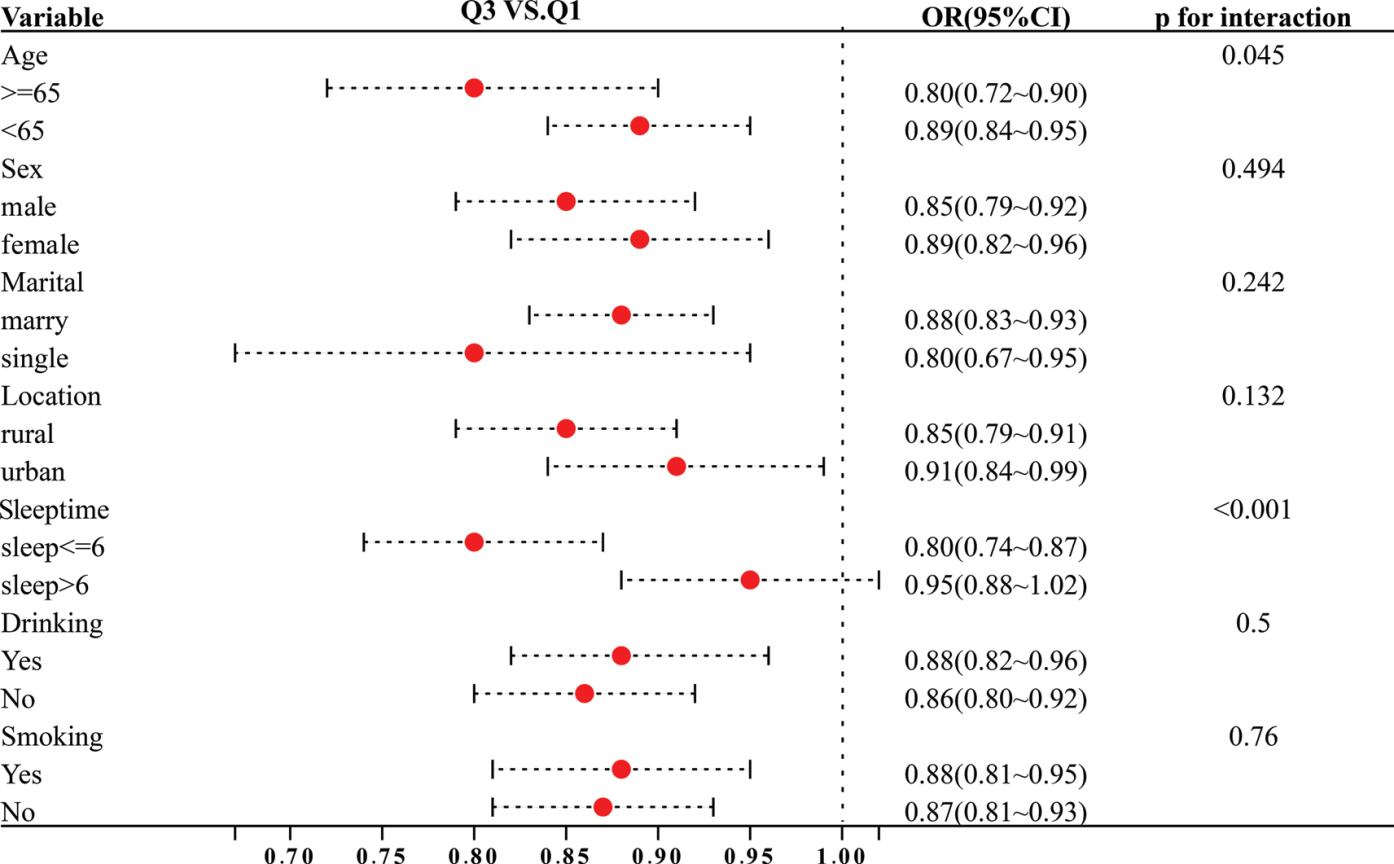
Forest plot of stratified analysis of the correlation between PEFpred% and frailty risk. OR = odds ratio, CI = confidence interval.

Given the possibility that the effect of PEFpred% may vary among different subpopulations, we further explored this association through subgroup analyses. To assess whether PEFpred% differentially impacts the risk of developing frailty across various populations, we conducted subgroup analyses based on age, sex, marital status, sleep duration, smoking, and alcohol consumption. The findings demonstrated a consistent effect of PEFpred% on frailty risk across these subgroups. Notably, the association was more pronounced in individuals aged ≥ 65 years, with an OR of 0.80 (95% CI: 0.72–0.90) compared to 0.89 (95% CI: 0.84–0.95) in younger individuals (*P* for interaction = .045). This suggests that older adults experience a more significant reduction in frailty risk with each unit increase in PEFpred%. Similarly, the relationship between PEFpred% and frailty risk was stronger in individuals reporting ≤ 6 hours of sleep, yielding an OR of 0.80 (95% CI: 0.74–0.87) compared to 0.95 (95% CI: 0.88–1.02) in those with longer sleep durations (*P* for interaction < .001). These results underscore the importance of personalized health intervention strategies aimed at enhancing PEFpred% and mitigating frailty risk, particularly among the elderly and those with insufficient sleep (Fig. 3).

### 3.4. Intermediation analysis

**Figure 4.**
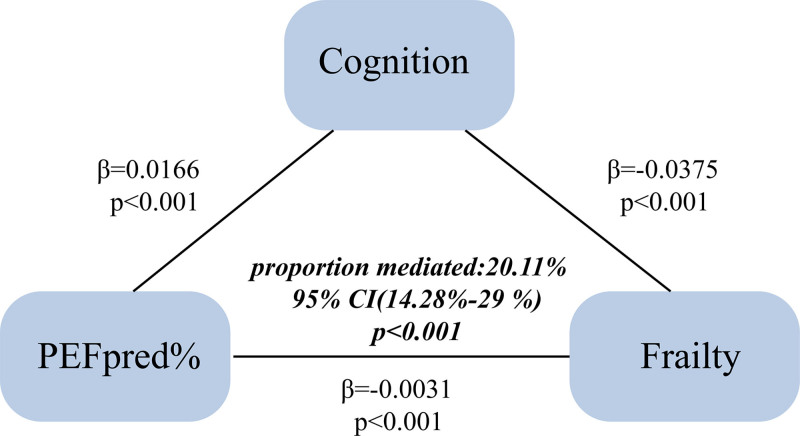
Cognitive function is a mediator in the path diagram of the relationship between lung function and frailty.

Additionally, we conducted a Bootstrap analysis to evaluate the mediating role of cognitive function in the relationship between PEFpred% and frailty, with the model pathway illustrated in Figure 4. In this analysis, PEFpred% served as the independent variable, cognitive function as the mediating variable, and frailty as the dependent variable. After adjusting for all covariates in the baseline data, the total effect of PEFpred% on frailty was estimated at -0.0031 (95% CI: -0.0038 to 0.0, *P* < .01). The direct effect of PEFpred% on frailty was -0.0025 (95% CI: -0.0038 to 0.0, *P* < .01), while the indirect effect through cognitive function was -0.0006 (95% CI: -0.0008 to 0.0, *P* < .01). This indicates that cognitive function partially mediates the relationship between PEFpred% and frailty, accounting for approximately 20.11% (95% CI: 14.28–29.00%, *P* < .001) of the total effect.

### 3.5. Development and validation of predictive models

**Figure 5.**
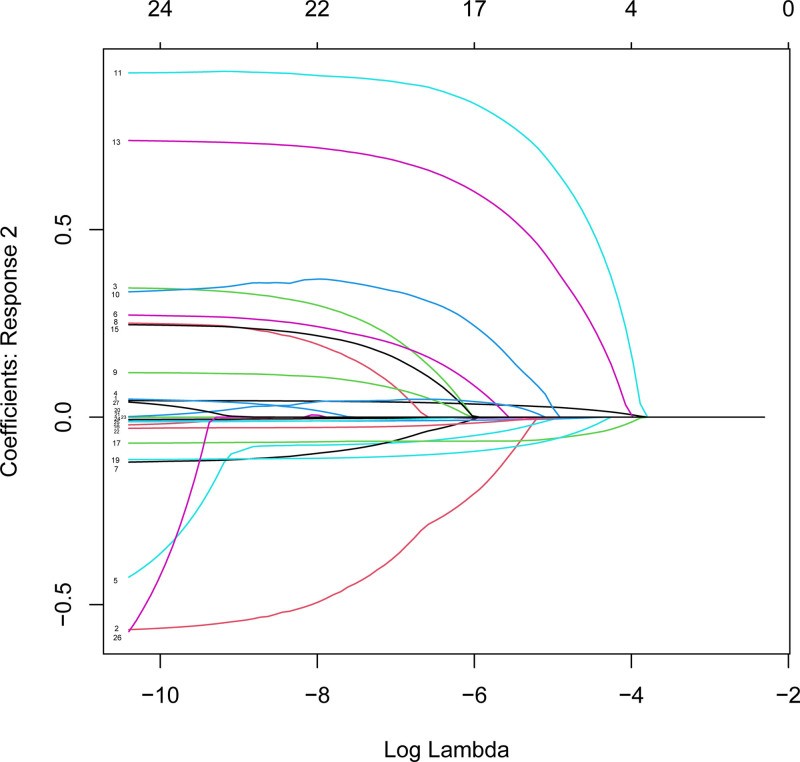
Generates coefficient profiles based on log(lambda) sequences and non-zero coefficients via optimal lambda. The optimal parameter (λ) in the LASSO model is selected by 10-fold cross-validation using the minimum criterion. LASSO regression model was used to select weakly correlated variables. LASSO = least absolute shrinkage and selection operator.

**Figure 6.**
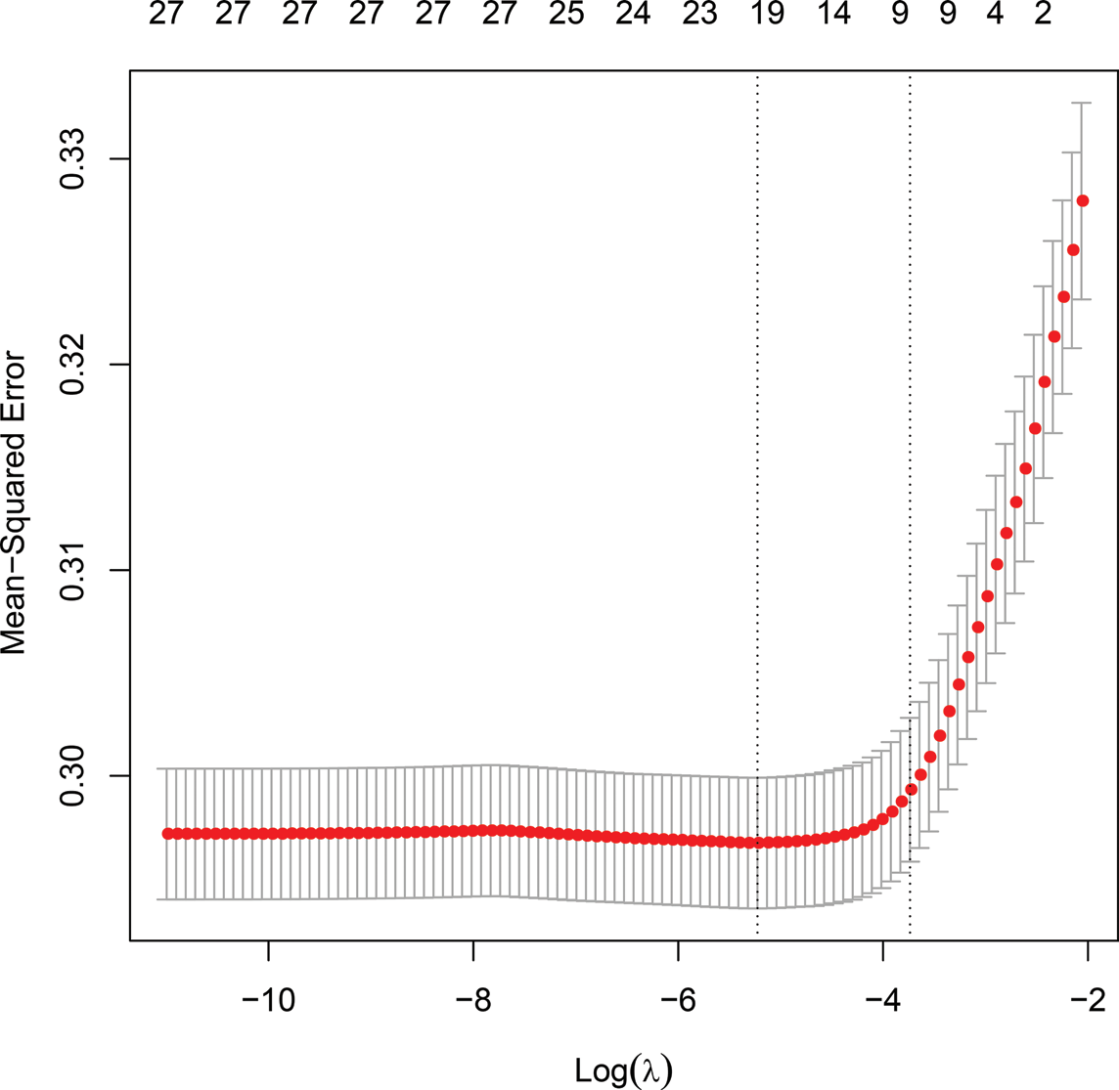
Plot of biased likelihood bias (binomial bias) with respect to log (lambda). LASSO regression model was used to select weakly correlated variables. LASSO = least absolute shrinkage and selection operator.

To enhance the prediction of frailty risk in the middle-aged and elderly population, we developed a predictive model. Participants were randomly assigned to a training set (60% of the total, comprising 3639 individuals) and a validation set (40%, consisting of 2426 individuals), with no significant differences observed between the 2 groups (*P* > .05). Least absolute shrinkage and selection operator regression analysis was employed, along with cross-validation, to identify the optimal λ value. A final λ of 1 standard error (λ.1se) was selected as the criterion for predicting the frailty latent factor (Figs. 5 and 6).

**Figure 7.**
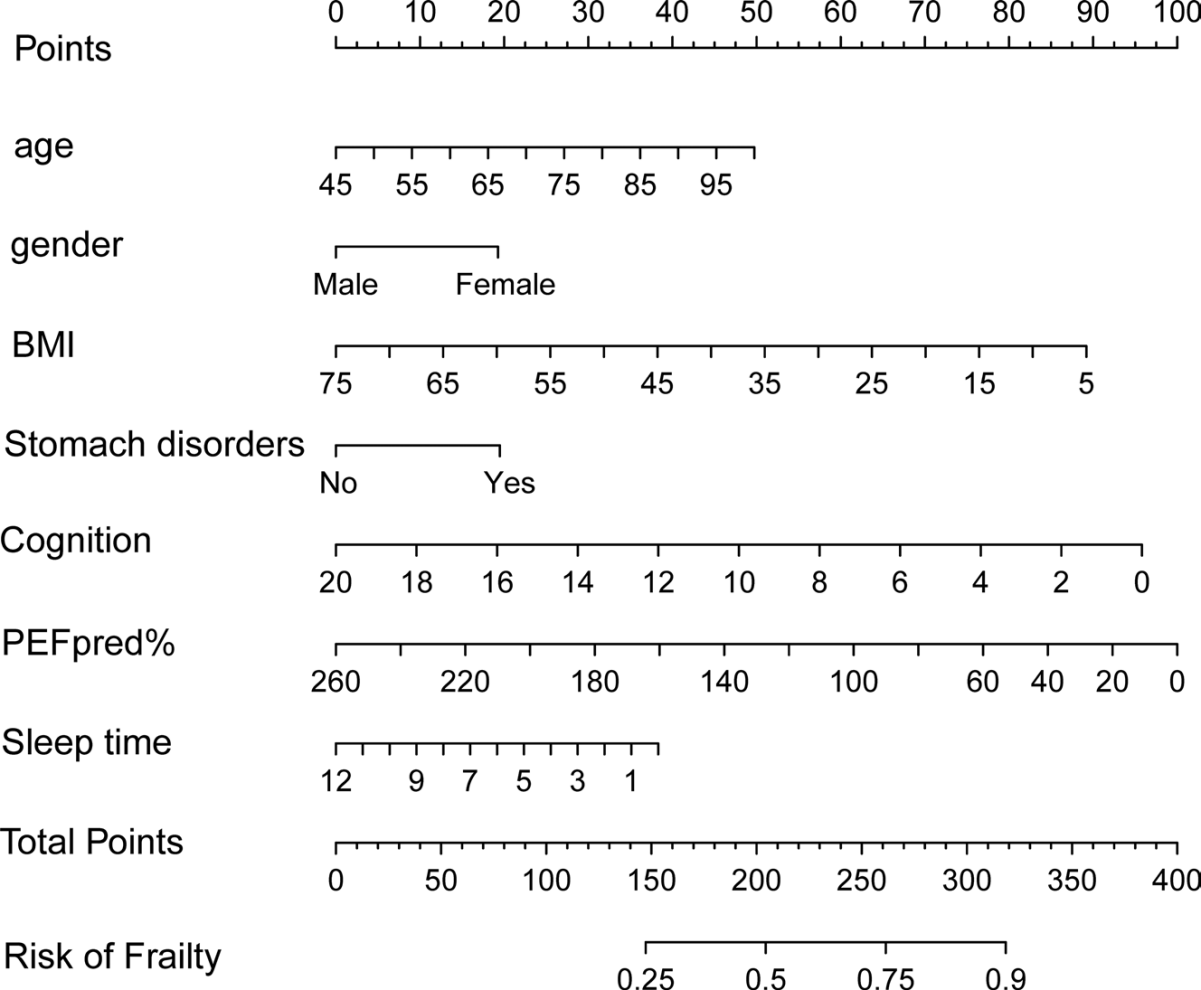
Nomogram representation for assessing frailty risk.

Based on a significance level of *P* < .05, several independent variables were included in the model: age (*P* = .002), gender (*P* = .043), BMI (*P* = .003), gastric disorders (*P* = .028), cognitive function (*P* = .023), PEFpred% (*P* = .001), and sleep duration (*P* = .006). Predictive models were constructed using multifactorial logistic regression and were visualized through a Nomogram, designed to quantitatively predict the risk of frailty (Fig. 7).

**Figure 8.**
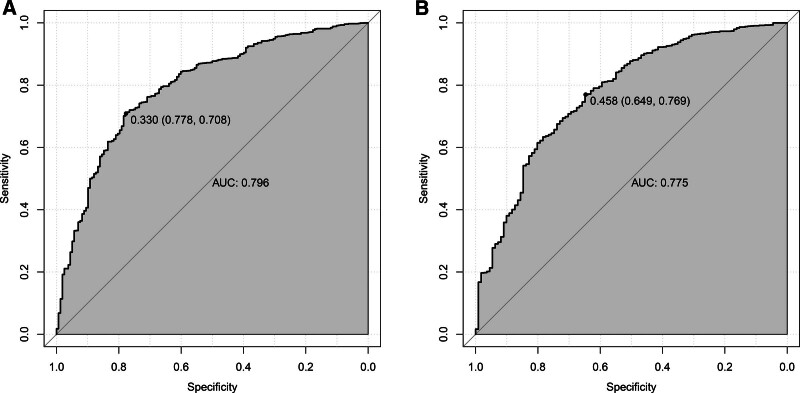
ROC curves of the nomogram generated from (A) the training dataset and (B) the validation dataset. ROC = receiver operating characteristic.

**Figure 9.**
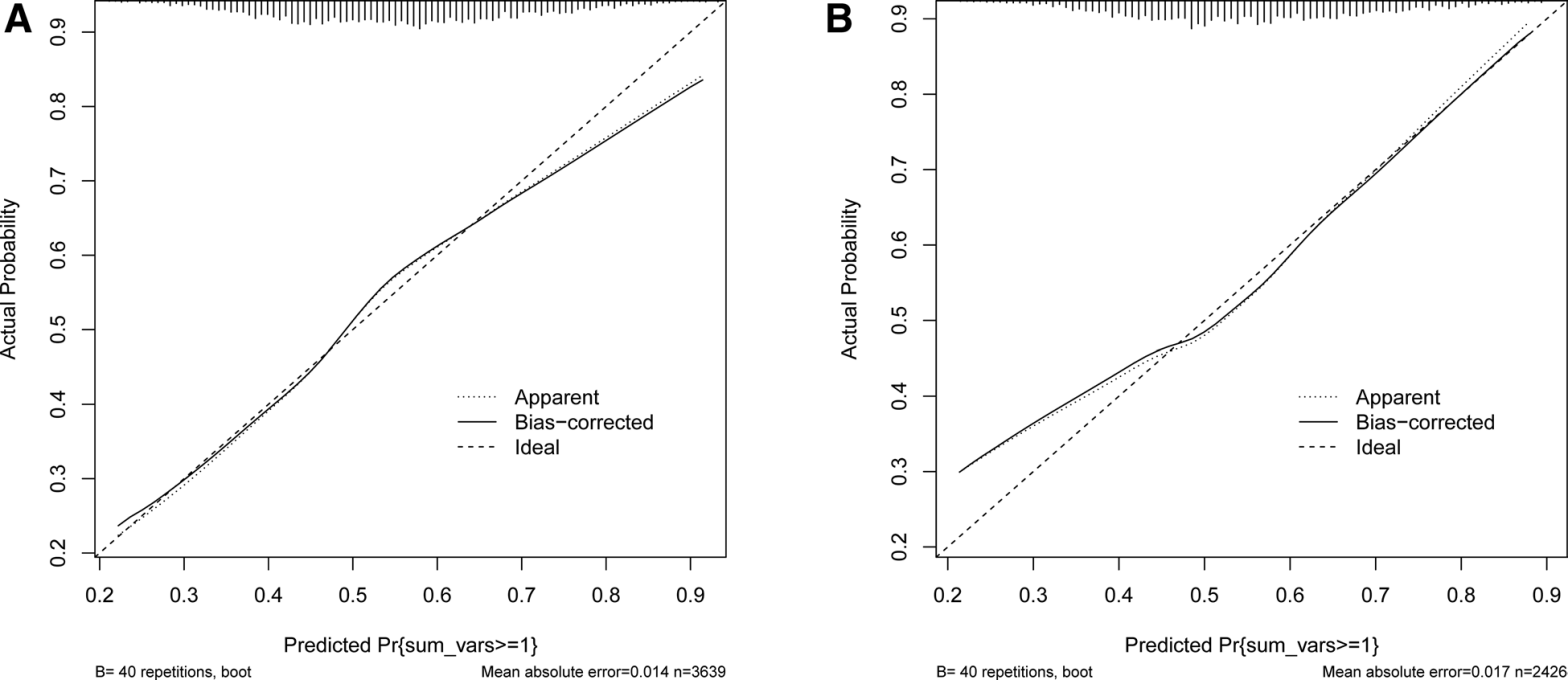
Calibration plots for the (A) training dataset and the (B) validation dataset.

Subsequently, to assess the predictive ability of the model, we constructed ROC curves. Meanwhile, to further verify the calibration of the model, the calibrate function was used to analyze the model. From the analysis results (Fig. 8), the model had a specificity of 0.778, a sensitivity of 0.708, and an AUC = 0.796 (95% CI: 0.7594–0.8324) in the training set, and a specificity of 0.649, a sensitivity of 0.769, and an AUC = 0.775 (95% CI: 0.7276–0.8216) in the validation set. These results suggest that the model has good discriminatory power and predictive value in distinguishing frail from non-frail patients. The calibration curve almost coincided with the Y = X line, indicating good agreement between the predicted and actual probabilities of the model pairs (Fig. 9).

## 4. Discussion

With aging, there is a notable decline in skeletal and respiratory muscle strength, accompanied by reductions in lung elastic retraction and thoracic compliance. This decline results in diminished respiratory function indices among middle-aged and older adults, including vital capacity, forced vital capacity, forced expiratory volume in one second, and PEF.^[13,27]^ PEF is particularly useful in assessing expiratory flow rates in chronic obstructive pulmonary disease and asthma patients and correlates with lung volume and respiratory muscle strength in healthy individuals.^[28]^ While declining PEF values may reflect normal age-related changes, significant reductions beyond a certain threshold are linked to various physical and psychological conditions.^[29]^ Although previous studies have explored the relationship between PEF and frailty, they have predominantly focused on South American and European populations.^[29,30]^ However, given the distinct differences in diet and exercise habits between the Chinese population and other regional groups, it is crucial to investigate the relationship between PEF and frailty within this demographic. Chinese diets, typically rich in vegetables and fruits with relatively low meat consumption, alongside traditional exercises such as taijiquan, may uniquely influence frailty and respiratory health outcomes.^[7]^ Most studies on PEF and frailty have been conducted outside East Asia, potentially overlooking these cultural factors. Therefore, research focused on the Chinese population is essential to better understand how PEF relates to frailty in this context, ultimately informing more culturally relevant health management strategies.

In this cross-sectional study of middle-aged and older Chinese adults, we identified a negative association between lung function (assessed by PEFpred%) and frailty risk. Specifically, higher baseline lung function was linked to a reduced risk of frailty, and this association was held after adjusting for various covariates. Dose–response analysis showed a nonlinear relationship, with a significant increase in frailty risk observed when PEFpred% fell below 80.03%. Subgroup analyses indicated that this inverse association was particularly strong among those aged ≥ 65 years and those who slept ≤ 6 hours, highlighting a heightened frailty risk in older and sleep-deprived individuals when lung function is compromised. Additionally, mediation analysis demonstrated that cognitive function mediated 20.11% of the effect of lung function on frailty, emphasizing the role of cognitive health in this relationship. Finally, we developed a predictive model integrating lung and cognitive function, which showed promising accuracy in frailty risk prediction among this population. These findings underscore the importance of lung and cognitive health in assessing and potentially mitigating frailty risk, particularly among older and sleep-deprived adults in China.

Although the mechanisms underlying the relationship between PEF and frailty are not fully understood, several plausible explanations have been proposed. Reduced pulmonary function may contribute to frailty through diminished oxygen delivery and chronic inflammation, which can impair muscle metabolism and promote sarcopenia in middle-aged and older adults.^[31,32]^ In contrast, cognitive function appears to play a more prominent mediating role. Existing evidence suggests a negative correlation between cognitive impairment and frailty, with better cognitive performance associated with improved health behaviors and reduced frailty incidence.^[33]^ Previous studies have also reported associations between grip strength and cognitive ability, indicating that muscle strength may serve as an intermediary linking respiratory and cognitive health.^[34]^ Moreover, PEF has been positively associated with cognitive function, and reduced lung function has been identified as an independent risk factor for cognitive decline.^[35]^ A possible explanation is that impaired pulmonary function leads to insufficient brain oxygenation, causing neuronal damage and neurotransmitter depletion, which contribute to memory loss and cognitive deterioration.^[36]^ Additionally, factors such as mental health, nutritional status, inflammation, and cardiovascular risk may modulate the interaction between cognitive impairment and frailty.^[37]^ The complex interplay of these multidimensional factors underscores the need for comprehensive interventions to address the multifaceted nature of frailty development and progression.

This study has several limitations. The cross-sectional design limits our ability to infer causality between pulmonary function and frailty. Although our findings demonstrate a negative association between PEF and frailty, a causal relationship remains speculative. However, prior Mendelian randomization studies support a negative association between PEF and frailty, suggesting a potential causal mechanism.^[38]^ Although the 2011 to 2012 CHARLS wave provided comprehensive baseline data, the use of a single wave limits the temporal applicability of our findings. Over the past decade, China has undergone substantial changes in public health and environmental conditions. For example, national smoking rates have declined due to strengthened tobacco control policies, while air quality has improved in many urban and rural areas.^[39,40]^ Furthermore, healthcare access has expanded, especially in older populations.^[41]^ These factors may influence both pulmonary function and frailty risk, potentially altering the strength or direction of the association observed in this study. Therefore, caution is warranted when extrapolating our findings to contemporary populations, and future studies using more recent or longitudinal data are needed to reassess the robustness of this relationship. Temperature changes affect PEF values.^[30]^ The data for this study were collected from communities in 28 provinces in China, which contained 150 county-level units and 450 village-level units. The large geographic spread made it difficult to standardize the temperature of the measurement environment. The variability in participants’ daily work and recreational activities results in differing individual activity thresholds. These thresholds influence the intensity and duration of physical activity, ultimately impacting the assessment of frailty status. Nevertheless, PEF remains a reliable indicator for predicting the onset of frailty in middle-aged and older adults. Recent studies involving stroke patients have demonstrated that respiratory muscle training can enhance swallowing and breathing parameters, thereby facilitating rehabilitation.^[42,43]^ In the future, this concept could be applied to healthy middle-aged and older adults to mitigate the risk of frailty by enhancing lung function. This approach offers a novel avenue for research and intervention aimed at improving the health and well-being of this demographic.

## Acknowledgments

The authors are grateful to all peers who provided their valuable support.

## Author contributions

**Conceptualization:** Sirui Zhou.

**Data curation:** Sirui Zhou.

**Formal analysis:** Sirui Zhou, Weijian Zhu.

**Methodology:** Ping Wang.

**Project administration:** Yulan Zeng.

**Software:** Weijian Zhu.

**Visualization:** Ping Wang.

**Writing – original draft:** Sirui Zhou.

**Writing – review & editing:** Ping Wang, Weijian Zhu, Yulan Zeng.

## Supplementary Material


