# Supplementary material for: Lung function and frailty: Dose–response relationship, mediation effects, and prediction model
Source: Medicine (Baltimore). 2025 Sep 12;104(37):e44529. doi: 10.1097/MD.0000000000044529 (PMC12440507; doi:10.1097/MD.0000000000044529)
Supplement: Supplementary file 1 [file medi-104-e44529-s001.docx]

Supplementary Table S1. Proportion and distribution of missing data across covariates. Data are based on participants aged ≥ 45 years at baseline (N = 17,314).

| Covariate | Non-missing | Missing | Missing (%) |
| --- | --- | --- | --- |
| Gender | 17314 | 0 | 0 |
| Marry | 17314 | 0 | 0 |
| Education | 17314 | 0 | 0 |
| BMI | 13274 | 4040 | 30.44 |
| Residence | 17001 | 313 | 1.84 |
| Drinking | 16866 | 448 | 2.66 |
| Smoking | 16882 | 432 | 2.56 |
| Hypertension | 16837 | 477 | 2.83 |
| Diabetes | 16866 | 448 | 2.66 |
| Cardiovascular disease | 16791 | 523 | 3.11 |
| Psycho | 16812 | 502 | 2.99 |
| Arthritis | 16848 | 466 | 2.77 |
| Stomach disorders | 16839 | 475 | 2.82 |
| Liver disease | 16762 | 552 | 3.29 |
| Chronic kidney disease | 16775 | 539 | 3.21 |
| Cognition | 12241 | 5073 | 41.44 |
| Sleep time | 15493 | 1821 | 11.75 |
| TC | 11285 | 6029 | 53.42 |
| TG | 11286 | 6028 | 53.41 |
| HDL | 11293 | 6021 | 53.32 |
| LDL | 11272 | 6042 | 53.6 |
| HbA1C | 11578 | 5736 | 49.54 |
| CRP | 11294 | 6020 | 53.3 |
| TyG | 11266 | 6048 | 53.68 |
| TyG_BMI | 9540 | 7774 | 81.49 |
| PEFpred% | 17314 | 0 | 0 |
